# A Review on Nutrients, Phytochemicals, and Health Benefits of Green Seaweed, *Caulerpa lentillifera*

**DOI:** 10.3390/foods11182832

**Published:** 2022-09-13

**Authors:** Nur Syakilla, Ramlah George, Fook Yee Chye, Wolyna Pindi, Sylvester Mantihal, Noorakmar Ab Wahab, Fazlini Mohd Fadzwi, Philip Huanqing Gu, Patricia Matanjun

**Affiliations:** 1 Faculty of Food Science and Nutrition, Universiti Malaysia Sabah, Jalan UMS, Kota Kinabalu 88400, Sabah, Malaysia; 2 Stemcell United Pte. Ltd., No.6, Defu Lane 2, Singapore 539466, Singapore; 3 Seaweed Research Unit, Universiti Malaysia Sabah, Jalan UMS, Kota Kinabalu 88400, Sabah, Malaysia

**Keywords:** *Caulerpa lentillifera*, sea grapes, nutrient content, nutrient composition, health benefits

## Abstract

*Caulerpa lentillifera* is a type of green seaweed widely consumed as a fresh vegetable, specifically in Southeast Asia. Interestingly, this green seaweed has recently gained popularity in the food sector. Over the last two decades, many studies have reported that *C. lentillifera* is rich in polyunsaturated fatty acids, minerals, vitamins, and bioactive compounds that contribute many health benefits. On the other hand, there is currently hardly any article dedicated specifically to *C. lentillifera* regarding nutritional composition and recent advancements in its potential health benefits. Hence, this study will summarise the findings on the nutritional content of *C. lentillifera* and compile recently discovered beneficial properties throughout the past decade. From the data compiled in this review paper, it can be concluded that the nutrient and phytochemical profile of *C. lentillifera* differs from one region to another depending on various external factors. As a result, this paper will offer researchers the groundwork to develop food products based on *C. lentillifera*. The authors of this paper are hopeful that a more systematic review could be done in the future as currently, existing data is still scarce.

## 1. Introduction

In 2019, Asia contributed to 97.4 percent of global seaweed production (99.1 percent from cultivation), with seven of the top ten producing countries located in Eastern or South-eastern Asia [1]. This indicates a significant regional imbalance in seaweed production which is largely influenced by the fact that seaweeds are a regular part of human diets in East Asia compared to elsewhere [2]. Seaweeds have been a food source since the fourth century in Japan and the sixth century in China. According to historical sources, people gathered macroalgae for sustenance as early as 500 B.C. in China and a thousand years later in Europe. People who lived near coastal areas preferred to consume seaweeds as a main dish or in soup [3]. Europeans usually consume smaller amounts of seaweed than Asians due to European regulations and dietary habits [4]. 

Although macroalgae intake is not as prevalent in Europe as in Asia, microalgae have acquired popularity because of their physiologically active components, earning them the reputation of "new superfoods" [5]. Between 1950 and 2019, global seaweed cultivation and production increased by a thousand-fold, with mainly brown seaweed (from 3.1 million tonnes to 16.4 million tonnes) and red seaweed cultivation (from 1 million tonnes to 18.3 million tonnes) being the main contributors [1]. However, the world cultivation of green seaweed decreased from 31,000 tonnes to 17,000 tonnes during the same period [2]. The 16,696 tonnes of green seaweeds grown in 2019 represented only 0.05 percent of the total seaweed production in the same year. Among the 16,696 tonnes produced were *Caulerpa* spp., *Monostroma nitidum*, *Enteromorpha* [*Ulva*] *prolifera*, *Capsosiphon fulvescens*, and *Codium fragile*, all of which are included in FAO’s Aquatic Sciences and Fisheries Information System. Out of 100 known *Caulerpa* species, only seven are utilised for human consumption globally, with *C. lentillifera* and *Caulerpa racemosa* dominating in this aspect [6]. Table 1 shows the global seaweed production and comparison by region in 2019.

**Figure 1:**
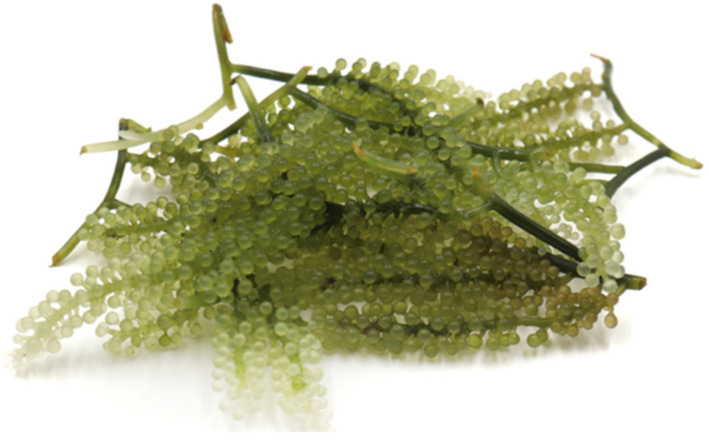
Fresh *C. lentillifera*.

Due to their grape-like appearance, they are commonly known as sea grapes or sea caviars. They are also known by different names in certain countries; most names directly translating the term "sea grape" into their vernacular. For instance, “nama” in Fiji, “bulung boni” in Indonesia, “umi budo” (海ぶどう) or “kumejima” in Japan, “bada podo” (바다 포도) in Korea, “lato”, “lelato”, or “ararosip” in the Philippines, “latok” in Malaysia, and “rong nho” or “rong nho biển” in Vietnam [7,8,9,10,11]. They usually inhabit sandy or muddy shallow sea bottoms [12]. *C. lentillifera* J. Agardh was originally described from the Red Sea coast [13]. It has been reported to be widely distributed in subtropical and tropical locations, such as the South China Sea, Southeast Asia, Japan, Taiwan, and Oceania, where it is directly consumed as a snack, in salads, and sushi, or in its salt-preserved form [14]. It has been described to have a salty taste and succulent texture. Figure 1 illustrates fresh *C. lentillifera*.

*C. lentillifera* is an alternative food that can also be used therapeutically. Over the years, it has gained popularity owing to its nutritional value, potential pharmacological benefits, and sustainability [15,16]). Within the past five years, several publications have reviewed various aspects of *Caulerpa* spp., such as its consumption, nutritional value, and farming [6], bioactive components and biotechnological applications [17], metabolite roles in cancer treatments [18], as well as its position as a functional food [11]. Only two publications had focused on reviewing the green algae genus *Caulerpa* in chemical composition, diversity, ecology, farming, pharmacological and industrial potential [10,19]). However, the review did not critically evaluate *C. lentillifera* specifically. To the best of our knowledge, no publication has focused solely on *C. lentillifera* in terms of its nutrient content and recent advances in potential health benefits that would make it suitable for pharmaceutical and nutraceutical use. Therefore, this article aims to review relevant literature in the past decade from reliable sources regarding *C. lentillifera* based on its nutrient content and beneficial properties. This paper would provide a good foundation for future researchers to develop functional food products utilising *C. lentillifera*. 

## 2. Nutritional Value of *C. lentillifera*

The proximate composition and the total dietary fibre content of *C. lentillifera* from different countries are shown in Table 2.

### 2.1. Carbohydrate and Fibres

The most abundant component in *C. lentillifera* are carbohydrates and dietary fibre. Dietary fibre is a complex mixture of carbohydrates and polymers in plants, including oligosaccharides and polysaccharides. Other non-carbohydrate components such as polyphenols, resistant proteins, saponins, and waxes may also be present [38]. However, these may vary even within its species [39]. For instance, although belonging to the same genus and family, *C. lentillifera* has higher carbohydrate content than *C. racemosa* [40]. *C. lentillifera* contains as low as 0.36% and as high as 72.9% carbohydrates in its dry matter (Table 2). Its dietary fibre content is approximately 17.5 to 36.7% in 100 g dried *C. lentillifera,* respectively. Water-soluble fibre content is approximately 2.45–17.21%. Water-soluble fibres are usually higher in red algae, around 15 to 22% in the dry matter, such as in *Chondrus crispus* (Irish moss) and *Porphyra/Pyropia* spp. (nori) [41,42].

In seaweed, soluble fibres can absorb water up to 20 times its volume [43]. This helps enhance the binding of water with food pellets in the gut and aids in stool bulking and shortening transit time in the colon; these act as positive factors that may prevent colon cancer [44]. In *Caulerpa* spp., soluble polysaccharides mostly consist of glucans and sulfated polysaccharides [19]. Sulfated polysaccharides from *C. lentillifera* have been reported to have physiological benefits, which will be discussed in the latter part of this review. Insoluble dietary fibres are generally not digested in the human gastrointestinal tract. Upon contact with water, they do not form gels but retain water in their structural matrix, increasing faecal bulk and accelerated intestinal transit [45]. Insoluble dietary fibres of *C. lentillifera* range from 15.75 to 28.98% (Table 2). However, *C. lentillifera* has lower dietary fibre content than other green seaweeds, such as *C. racemosa* and *Ulva reticulata,* at 65.7% and 64.9%, respectively [40,46,47]. In adults, high consumption of dietary fibre, particularly fermentable fibres, has been linked with increased short-chain fatty acid (SCFA) contents in the stool [44,48].

### 2.2. Protein and Amino Acids

With increased population growth and demand for protein, seaweeds are plausibly viable and sustainable protein sources due to their low environmental impact and fast-growing rate. Furthermore, the protein content of whole algae is very high compared to common food staples such as cereals, legumes, and nuts [49]. With its versatility and simplicity of usage, whole algal protein has the potential to be a tremendous whole-food protein source, as well as a great way to supplement protein-deficient diets [50]. When comparing the protein contents, the levels of proteins are higher in Rhodophyta (red), followed by Chlorophyta (green), and Ochrophyta (brown) [51,52]. The protein content of *C. lentillifera* ranged from 0.43 to 19.38% in various countries (Table 2). The wide difference and instability of the protein content could be affected by various external factors, such as water temperature, season, geography, weather, and other factors [46]. It was reported that protein content in seaweed was higher in winter than in autumn and summer [53,54].

The protein quality depends on the presence and quantity of essential amino acids. Amino acids are the building blocks that form proteins bound together via peptide bonds formed between the carboxyl group of an amino acid and the amino group of the next amino acid in line [55]. *C. lentillifera* are considered to have high-quality proteins as the essential amino acids present and were close to egg and soya protein content [54]. Except for tryptophan, almost all essential amino acids (EAA) are present. Their amino acid profile is dominated mainly by leucine, valine, aspartic acid, glutamic acid, and glycine. The major amino acids in seaweed proteins are aspartic and glutamic acid, which contribute to the umami flavour [56]. The amino acid profile of *C. lentillifera* is shown in Table 3.

### 2.3. Minerals

Minerals absent from freshwater algae and terrestrial crops are mostly available in seaweeds [57]. Minerals are essential and required in certain amounts for the normal metabolic functioning of the human body [58]. The mineral element found present in *C. lentillifera*, including essential minerals and toxic minerals, are presented in Table 4. The mineral content varies due to the phylum or class of the seaweed and geographical origin, along with seasonal, environmental, and physiological variations [39]. 

Essential minerals crucial for human wellbeing include Ca, Cu, Fe, Mg, Zn, K, Na, P, Se, Mn, Cr, and I [60,61]. On the other hand, toxic minerals such as Al, As, Cd, Hg, and Pb do not possess any benefits to humans but cause detrimental effects, which are present in *C. lentillifera* [62,63]. As stated in Table 4, Na, Mg, K, Ca, and Mn has a wide range of concentrations, among all mineral elements, with the highest concentration in Na (14.90–130,794 mg/100 g). For Mg, the highest concentration value was around 8126.59 mg/100 g (in China) to 10,663 mg/100 g (in Vietnam). The highest concentration value of K and Mn were found in *C. lentillifera* from China, 4967.34 mg/100 g and 1341.07 mg/100 g. 

The calcium content in *C. lentillifera* is comparable to common foods such as milk products, meat, fish, poultry, and legumes. For instance, the highest concentration value found in *C. lentillifera* was 8137 mg/100 g (in Vietnam) which is 4 times higher than the calcium content in high calcium milk powder, 2000 mg/100 g [64,65]. Iodine and iron are important to the human diet, both of which can be found in high concentrations in seaweeds, including *C. lentillifera* [66]. Insufficiency and deficiency of iodine could lead to goiter and hypothyroidism [65]. Although the iodine content in *C. lentillifera* is relatively low compared to in other green seaweed such as *Ulva clathrata* [67], it can be considered a cheap and reasonable option to fulfil the minimum iodine required needed by the body [65,68]. 

The deficiency of iron is a major health problem worldwide. The root of this problem is caused by prolonged inadequate intake due to low bioavailability in the diet. Especially during the period of growth and chronic blood loss, the increase in iron requirement may also cause iron deficiency [65]. The consumption of *C. lentillifera* could be a potential iron supplement to combat iron deficiency. However, it is difficult to generalise or conclude whether the mineral contents in *C. lentillifera* is high or low, as different sampling region have greatly varied environmental conditions [19]. From the compiled data in Table 4, it can be concluded *C. lentillifera* are rich in minerals that meet the requirement of the human body. However, the Na/K ratios need careful consideration, as it has been reported to be higher than in other seaweeds such as *Sargassum polycystum* and *Eucheuma cottonii* [24]. If the Na/K ratio is too high, it is detrimental to the sodium to potassium balance in the human body, which can result in cardiovascular diseases. A simple desalting operation, such as soaking, is recommended before eating [32].

### 2.4. Lipids

*C. lentillifera* are significantly low in lipid content ranging from 0.05 to 14.0% in dry weight. Despite low lipid composition, *C. lentillifera* has raised interest due to a high content of long-chain polyunsaturated fatty acids (PUFAs) and carotenoids [19,41]. Compared to terrestrial vegetables, *C. lentillifera* contain significantly higher levels of polyunsaturated fatty acids, which act as strong antioxidants, such as ω-3 and ω-6 [69], which have various roles in the prevention of cardiovascular diseases, osteoarthritis, and diabetes [70]. The fatty acids profile of *C. lentillifera* are shown in Table 5. The ω-3 and ω-6 PUFAs, particularly linoleic acid (18:2ω6) and 𝛼-linolenic acid (18:3ω3), cannot be synthesised by most heterotrophic organisms and can only be obtained through dietary intake [32,71]. All these PUFAs can be found in *C. lentillifera*, with 𝛼-linolenic acid (18:3ω3) being the most abundant [71]. The fatty acid compositions of *C. lentillifera* are as tabulated in Table 5.

### 2.5. Vitamins

Seaweeds are known to be a good source of both water-soluble and fat-soluble vitamins. The requirements of vitamin A, B2, B12, and two-thirds of vitamin C in the human body could be fulfilled by consuming 100 g of seaweed [72,73]. Table 6 indicates the vitamin content found in *C. lentillifera* with its daily RNI and daily UL for comparison purposes. 

Water-soluble vitamin C is the most abundant in *C. lentillifera,* the major contributor to its antioxidant properties, with concentrations ranging from 0.028–274 mg/100 g. Among other seaweed groups, *C. lentillifera* is generally rich in B group vitamins [74]. Vitamin B1, B2, and B3 were present in *C. lentillifera* in trace amounts; however, the amount detected still exceeded the recommended daily intake. The total amount of vitamin B2 in *C. lentillifera* is considerably higher than in various legumes, including chickpeas, lentils, red and black grain, and soya beans, which contain relatively high riboflavin levels of around 0.2–0.5 mg/100 g [64]. Recent data on the riboflavin content of selected commercial rice, such as fragrant rice, basmati rice, and Siam rice, showed that all varieties contain 0.06 mg riboflavin per 100 g [75]. The amount of B3 in *C. lentillifera*, 1.9–200 mg/100 g, was also higher than that of *Ulva fasciata*, 1.02 mg/100 g, and *E. flexuosa*, 0.98 mg/100 g [76].

### 2.6. Pigments

The most abundant pigments in the *Caulerpa* species are chlorophylls, mostly composed of chlorophyll a and b [77]. Chlorophylls have an antioxidant property that makes them useful nutritional and a health supplement [78]. Chlorophylls available in our diet are obtained via the consumption of green vegetables. Several studies have demonstrated that chlorophylls and their degradation products have anti-proliferative and anticancer properties [41,79]. Carotenoids which are tetraterpenoid pigments are also found in *C. lentillifera*. Most carotenoids were present in seaweeds, such as α- and 𝛽-Carotene, lutein, and zeaxanthin, in which all except α-carotene were detected in *C. lentillifera*, as shown in Table 7. 

*β*-carotene is a precursor of vitamin A (retinol), an essential vitamin that promotes a healthy immune system, good skin, and eye health [78]. *β*-carotene also has antioxidant properties that protect the body from free radicals produced by oxidation of other molecules [81]. Carotenoids like lutein and zeaxanthin prevent the progress of age-related macular degeneration [56,82]. Caulerpin is a bis-indole alkaloid found in genus *Caulerpa* [83]. In *C. lentillifera*, it is found present at concentrations of 25.79–33.59 μg/g. This compound contributes to some of its reported therapeutic activities. For instance, caulerpin isolated from *Caulerpa taxifolia* showed anti-diabetic properties [84], whereas caulerpin sourced from other *Caulerpa* spp. demonstrated potential anti-inflammatory and anti-nociceptive properties [85]. The health benefits of caulerpin extracted from *C. lentillifera* will be covered in the next section of this review paper.

## 3. Health Benefits of *Caulerpa lentillifera*

**Figure 2:**
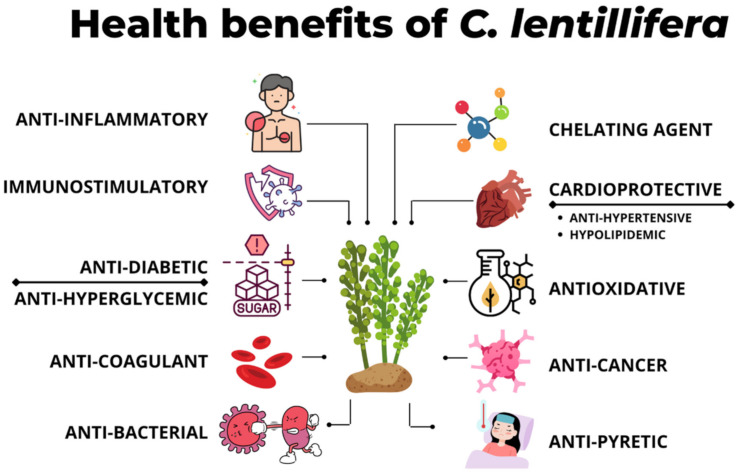
Overview of health benefits reported of *C. lentillifera*.

*C. lentillifera* has been discovered to have health-related functionalities that could be used for medical treatment and prevention, as illustrated in Figure 2. Table 8 highlights the documented health benefits of *C. lentillifera* in the literature throughout the previous decade.

### 3.1. Cardioprotective

#### 3.1.1. Anti-Hypertensive

Cardiovascular disease (CVD) is one of the noncommunicable diseases that is the most probable cause of mortality globally, besides cancer, diabetes, and chronic respiratory diseases, among people between the age of 30 to 70 years old [86]. Hypertension, or an increase in arterial blood pressure, is a major risk factor for CVD, affecting 15% to 20% of the world population [87,88,89,90]. One of the most important therapeutic approaches in managing hypertension is the inhibition of the Angiotensin-converting enzyme (ACE), as demonstrated in many clinical trials [8,87]. ACE inhibitors block the conversion of angiotensin I to angiotensin II, resulting in blood vessel relaxation and decreased blood pressure [89]. Pharmaceutical manufacturers have commercialised many ACE inhibitors to lower angiotensin II concentrations for the treatment of hypertension; however, these drugs possess adverse side effects, emphasising the need for developing natural food-derived inhibitors with fewer undesirable side effects [91].

In an in vitro study, protein hydrolysates from *C. lentillifera* were obtained using four different enzymes: α-chymotrypsin; pepsin; thermolysin; trypsin [92]. All hydrolysates obtained have demonstrated ACE-inhibiting properties, with the thermolysin hydrolysate showing the highest inhibition with 90.64% inhibition at a dose concentration of 1 mg/mL [92]. From their investigation, they concluded that the bioactive components responsible for this inhibitory activity were oligopeptides, FDGIP (FP-5), and AIDPVRA (AA-7). Although this is the first reported study utilising protein peptides from *C. lentillifera*, there are many other similar studies sourcing protein peptides from different seaweed species such as *Undaria pinnatifida*, *Saccharina japonica*, *Sargassum fusiforme*, *S. maclurei (Ochrophyta)*, *Gracilariopsis lemaneiformis*, *Mazzaella japonica*, *Palmaria palmata*, *Pyropia/Porphyra* spp., *Bangia fusco-purpurea (Rhodophyta)*, *Ulva rigida*, *U. chlatrata*, and *U. intestinalis (Chlorophyta)* [93,94,95,96,97,98,99,100,101,102,103,104,105,106,107,108].

**Table 8:** Health benefits reported in *Caulerpa lentillifera*.

Health Benefits Reported	Extract from *C. lentillifera*	Model of Study	Dosage	Reference
Anti-hypertensive	Dried *C. lentillifera powder*	α-chymotrypsin, pepsin, thermolysin, and trypsin	1 mg/mL	[92]
		Male Wistar rats (8–9 weeks old; 338 g)	5% dw	[24]
Anti-hyperlipidaemic	Dried *C. lentillifera*	Male Sprague-Dawley rats (10 weeks old, 260–300 g)	5 g/100 g	[24]
	*C. lentillifera* extract	Male rabbits	10, 158.5 and 39,810.70 mg/kg bwt	[109]
	Aqueous extract	Porcine pancreatin	5 mg	[14]
Anti-bacterial; anti-microbial	*C. lentillifera* extracts	*E. coli*, *S. aureus*, *Streptococcus* sp., *Salmonella* sp.		[26]
	Caulerpin	*E. coli*, *S. aureus*, *Streptococcus* sp., *Salmonella* sp.		
	Methanolic extract	*Staphylococcus aureus, Streptococcus mutans*	25–250 mg/mL	[110]
	Methanolic extract	Methilin-resistant *Staphylococcus* *aureus* (MRSA), *Escherichia coli* K1	250 µg/mL	[111]
Anti-tumour; anti-cancer; anti-proliferative; apoptotic	ß-1,3-Xylan	Human breast cancer cells, MCF-7 cells	1–2 mg/mL	[112]
	Ethanol-hexane Extract	A172 Human glioblastoma cells	200–1000 µg/mL	[113]
Anti-coagulant	ß-1,3-Xylan	Rabbit plasma	1, 3, 5, 10 and 20 mg/mL	[114]
	Aqueous extract	Male albino rabbits (4–6 months old, 1.0–1.25 kg) and canine blood samples	3 mg/mL	[115]
Anti-hyperglycaemic	Hydroethanolic Extract	Male albino mice	10 and 50 mg/kg	[116]
	Freeze-dried aqueous extract	Male BALB/c mice (6 weeks old)	600 and 1000 mg/kg bwt	[34]
Anti-diabetic	Ethanolic extract	Rat insulinoma cells (RIN), 3T3-L1 cells	1000 µg/mL	[117]
			10–25 µg/mL	
		L6 rat skeletal muscle cells	250 µg/mL	[118]
		6-week-old db/db male mice	250 and 500 mg/kg	
		Rat insulinoma (RIN)-m5F cells	250, 500, and 1000 μg/mL	[119]
Anti-inflammatory	*C. lentillifera* extracts	Murine macrophage RAW 264.7 cells	50 µg/mL	[26]
	Caulerpin	Murine macrophage RAW 264.7 cells	25, 50, 100 µg/mL	
	Sulphated polysaccharides	HT29 colonic carcinoma cells	50, 100, 200, 300 and 400 µg/mL	[120]
Antioxidative	Freeze-dried aqueous extract	Male BALB/c mice (6 weeks old)	600 and 1000 mg/kg bwt	[24]
Anti-pyretic	Aqueous extract	Adult male mice (24–30 g)	500 mg/kg bwt	[121]
Chelating agent	Aqueous extract	Male Sprague Dawley rats (4 weeks, 150–180 g)	500 mg/kg bwt	[122]
Immunostimulatory	Sulphated polysaccharides	Murine macrophage RAW 264.7 cells	1–5 µg/mL	[123]
	Xylogalactomannnans	Murine macrophage RAW 264.7 cells	50–800 µg/mL	[124]
	Polysaccharides	Mouse RAW264.7 cells	6.25, 12.5, 25 and 50 μg/mL	[125]
	In vitro fermented culture	60 cytoxan (CTX) induced immunosuppressed male BALB/c mice; 20 g	25, 50, and 100 mg/kg bwt	[126]

#### 3.1.2. Anti-Hyperlipidaemic

Lipids are one of the important nutrients required by the human body. High intake of lipids, however, could lead to obesity and hyperlipidaemia [127]. Hyperlipidaemia is characterised by a rise in blood total cholesterol (TC), low-density lipoprotein (LDL), very low-density lipoprotein (VLDL), and a reduction in high-density lipoprotein (HDL) [128]. It is a major cardiac risk factor, and it has been linked to an increased risk of cardiovascular disease in these patients [129]. Although the current drugs used in medical practices are very effective in lowering LDL levels, these drugs do have side effects which cause patients to seek treatments using safe and naturally derived drugs. At present, much research has evaluated seaweed-polysaccharides effect in lowering blood lipid levels. The evaluations are mainly conducted based on in vivo and in vitro experiments. In an in vitro experiment, mice were fed a high cholesterol and high fat (HCF) diet to establish a hyperlipidaemic model study. Then, the mice were treated with seaweed polysaccharides in which their blood lipid-related factors, lipase inhibition rate, and bile salts binding capacity were determined. 

In an in vivo study, treatment of HCF rats with 5% dried *C. lentillifera* for 16 weeks significantly lowered their body weight by 39.5%, increased HDL levels by 48.7%, reduced TC by 18.4%, LDL by 34.6%, and triglycerides levels by 33.7%, and lowered lipid peroxidation level by 9%, erythrocyte glutathione peroxidase level by 31.8% and catalase level by 3.14%, compared to the corresponding levels in HCF rats [24]. Similar findings were obtained in another in vivo study where a decrease in total cholesterol levels was observed among hypocholesterolaemia-induced male rabbits administered with crude *C. lentillifera* extract [109]. The anti-hyperlipidaemic effects of different polysaccharide fractions of *C. lentillifera* extract, i.e., WCLP25, WCLP40, WCLP55, WCLP70, and WCLP85, were assessed in a simulated bile acid-binding experiment. From the in vitro experiment conducted, they found out that WCLP-55 and WCLP-70 are potentially applicable for lowering blood lipids as these fractions have significantly higher binding capacities for cholic acid, deoxycholic acid, glycocholic acid, and taurocholic acid) [14]. Other polysaccharides sourced from different seaweed species demonstrated hypolipidemic properties, such as *Sargassum polycystum*, *Enteromorpha prolifera*, *Monostroma nitidum*, *Sargassum fusiforme*, and *Ulva pertusa* [130,131,132,133].

### 3.2. Antibacterial and Antimicrobial Activity

Antimicrobials are compounds that kill or hinder the growth of microbial pathogens, respectively, whereas antibiotics and antifungals are compounds that help kill them [130]. Antimicrobials primarily impact microbial cells, targeting the phospholipid bilayer of the cell membrane, destroying enzyme systems, and altering the bacteria’ genetic material [134]. Secondary metabolites from seaweeds such as polyphenols or other bioactive compounds can disrupt the permeability of the microbial cell, and interfere with membrane function, thus, consequently causing cell apoptosis [135]. In a study, the antibacterial potential of *C. lentillifera* extracts and caulerpin against four common food microbial pathogen strains, i.e., *E. coli*, *Salmonella* sp., *Streptococcus* sp., and *Staphylococcus aureus,* were evaluated [26]. The seaweed extract was found to demonstrate antimicrobial activities in all test organisms with the range of minimum inhibitory concentrations of 136.5, 125.25, 175.25, and 140.50 MIC/mg mL^−1^ in *E. coli*, *Staphylococcus aureus*, *Streptococcus* sp., and *Salmonella* sp. respectively. As for the caulerpin extract, it demonstrated antimicrobial activities in all test organisms with minimum inhibitory concentrations of 5.25 MIC/mg mL^−1^ in *E. coli*, *S aureus*, and *Salmonella* sp., and the lowest in *Streptococcus* sp., 15.50 MIC/mg mL^−1^.

### 3.3. Anti-Cancer

Existing anticancer medicines are often nonspecific, have side effects, or are exceedingly expensive; therefore, the search for improved therapeutics continues, with a particular focus on naturally occurring compounds. In an *in-vitro* study, it was discovered that ß-1,3-Xylan extracted from *C. lentillifera* inhibited the growth of MCF-7 human breast cancer cells and triggered chromatin condensation, degradation of poly ADP-ribose polymerase (PARP), and activation of caspase-3/7, indicating that it promoted death in these cells (MCF-7 cells) [123]. In other in vivo and in vitro experiments utilising bioactive compounds extracted from *Sargassum wightii and E. cottonii,* similar findings have been observed [136,137,138]. Despite similar outcomes observed in the previously mentioned experiments, different molecular mechanisms may occur as each compound, i.e., phloroglucinol, fucoxanthin, and fucoidan, have different action mechanisms, such as anti-angiogenic, antioxidative, anti-metastasis, anti-proliferative, and pro-apoptotic [139]. Recently, a fascinating discovery by Tanawoot and others [113] revealed that A147 glioblastoma cells treated with ethanol-hexane seaweed extracted from *C. lentillifera* demonstrated a drastic drop in cells viability and inhibited glioblastoma cell cycle progression in a high dose-dependent manner. The seaweed extracts also promoted the apoptosis of A147 cells.

### 3.4. Anti-Coagulant

Anti-coagulant is a key agent for preventing thrombosis, with heparin being the most often used commercial antithrombotic medication [140]. An *in-vitro* study using rabbit plasma treated with different concentrations of ß-1,3-Xylan, a polysaccharide compound extracted from *C. lentillifera,* has demonstrated prolonged activated partial thromboplastin time (aPTT). A similar result was observed in another study by Arenajo and colleagues, whereby an aqueous extracted from *C. lentillifera*, the concentration of 3 mg/mL tested in male albino rabbits and canine blood samples was able to prolong the clotting time dose-dependently [115].

### 3.5. Anti-Diabetic and Anti-Hyperglycaemic 

Diabetes is a type of metabolic disorder considered a chronic health problem globally. This disorder occurs when the pancreas does not produce enough insulin, Type-1 Diabetes, or when the body cannot use the insulin effectively upon production, Type-2 Diabetes [86]. Seaweeds have been widely used for anti-diabetic treatments [34,141]. Ethanolic extracts of *C. lentillifera* have been assessed both in in vivo and in vitro experiments resulting in positive anti-diabetic effects. An in vitro experiment conducted by *Sharma* and others [117] in rat insulinoma cells (RIN), 3T3-L1 cells exhibited a decrease in dipeptidyl peptidase-IV and α-glucosidase enzyme activities at 1000 µg/mL dosage concentration, whereas, at a 10–25 µg/mL dose concentration, ethanolic *C. lentillifera* extract showed inhibited cell death and iNOS expression in interleukin- 1β and interferon-γ induced RIN cells. Enhanced insulin secretion in pancreatic β-cells and increased insulin sensitivity and glucose uptake in 3T3-L1 adipocytes were observed at 10–25 µg/mL dose concentration administered [117]. According to the American Diabetes Association, hyperglycaemia refers to a high blood glucose level where the blood glucose is greater than 125 mg/dL while fasting with greater than 180 mg/dL 2 h postprandial. When 10 mg/kg and 50 mg/kg of hydroethanolic extract from *C. lentillifera* were introduced to male albino mice, it induced significant antihyperglycemic effects in the fasting state and 2-h postprandial loading in a dose-dependent manner [116]. Similarly, 600 and 1000 mg/kg of freeze-dried aqueous extract from *C. lentillifera* were orally administered to male BALB/c mice, which showed improved plasma glucose, insulin, and homeostasis model assessment-insulin resistance (HOMA-IR) levels after 6 weeks [34].

### 3.6. Anti-Inflammatory 

Inflammation is a natural defensive response to harmful stimuli such as irritants, pathogens, or damaged cells. Microbial infections, tissue stress, and some traumas are all examples of threats that trigger inflammation, which is frequently followed by symptoms such as fever, redness, swelling, and pain [142,143]. The inflammatory response is characterized by the overproduction of proinflammatory cytokines such as tumour necrosis factor-alpha (TNF-𝛼), interleukin (IL) (IL-6 and IL-1), prostaglandin E2 (PGE2), nitric oxide (NO), and increased production of reactive oxygen species (ROS) [144]. Increased inducible nitric oxide synthase (iNOS) and cyclooxygenase-2 (COX-2) activity is often linked to increasing NO and PGE2 production [141]. Although *C. lentillifera* has been a research subject regarding its health benefits, not many reports are found pertaining to its anti-inflammatory activity except for an *in-vitro* study by Nagappan and Vairappan [26] and Sun and others [120]. 

Using murine macrophage RAW 264.7 cells as the model of study, Nagappan and Vairappan found that the *C. lentillifera* extracts and caulerpin, an active ingredient extracted from *C. lentillifera*, when subjected to the RAW 264.7, did not release lactate dehydrogenase (LDH) and suppress the NO production. They also found that the production of nitrite and proinflammatory cytokines, TNF- 𝛼 and IL-6, were lowered in a dose-dependent manner. Meanwhile, Sun and others studied the anti-inflammatory activity of *C. lentillifera* by treating HT29 colonic carcinoma cells that have been lipopolysaccharides (LPS) induced with four different fractions of sulphated polysaccharides extracts, i.e., CLGP1, CLGP2, CLGP3, and CLGP4 [120]. From their research, they concluded that LPS-stimulated HT29 cells treated with CLGP4, *C. lentillifera* polysaccharides demonstrated a powerful inhibition of the production of interleukin-1ß (IL-1ß) as well as the tumour necrosis factor (TNF-α), significantly reduced the mucin2 production in a dose-dependent manner. Among all sulphated saccharides, CLGP4 had the best anti-inflammatory effect in vitro.

### 3.7. Antioxidant 

Antioxidant phytochemical compounds can scavenge reactive oxygen and nitrogen species (ROS and RNS) in the human body, slowing or preventing the onset of oxidative stress-related diseases such as cancers, cardiovascular diseases, delayed sexual development, kidney and liver diseases, neurological disease, respiratory diseases, and rheumatoid arthritis [145,146,147,148]. One of the most prominent health benefits of *C. lentillifera* is its antioxidant properties. Among all solvent extractions used, it can be observed that ethanolic and methanolic extracts showed antioxidant activity in various tests, as tabulated in Table 8. The different levels of activities exhibited in these antioxidant tests could be correlated to the polarity of the solvent extraction used. However, *C. lentillifera* extracted using water, i.e., the most polar organic solvent, showed a much lower antioxidant activity than when methanol and ethanol were used. This could be due to the dependency of the antioxidant activity on the synergistic effects of the extraction solvent used [149]. In a recent in vivo study, freeze-dried aqueous extract of *C. lentillifera* was observed to reduce antioxidative stress in diabetic mice and prevent male reproductive system dysfunction [34]. Overall, *C. lentillifera* can be seen to have high phenolics and flavonoid content, good scavenging and reducing properties, and high Trolox equivalent antioxidant capacity, as shown by the data tabulated in Table 9.

### 3.8. Anti-Pyretic

Anti-pyretic or analgesic helps prevent or alleviate fever. Fever is a common medical symptom characterised by a 37.2 °C fever induced by infection or inflammation. Three of the most common over-the-counter synthetic anti-pyretic drugs are aspirin, acetaminophen (paracetamol), and ibuprofen [152,153]. These drugs reduce fever by inhibiting the expression of cyclooxygenase (COX-2), which results in the production of prostaglandins [153,154]. However, these synthetic anti-pyretic drugs raise considerable concerns since they can lead to a few adverse pharmacological [155,156]. Hence, there is a global need for drugs produced from natural resources that have a minimal detrimental impact on human health. *C. lentillifera* has been evaluated in an in vivo study to determine whether it could potentially be used as an anti-pyretic agent [121]. 500 mg/kg per body weight of aqueous extracts from the seaweed were administered orally to adult male mice with body weights ranging around 24 to 30 g. From their experiment, they concluded that *C. lentillifera* showed a significant anti-pyretic effect as the rectal temperature of mice with fever decreased by 1.15 °C, 5 h after consumption of aqueous seaweed extract as compared to the control given 10 mg/kg of acetaminophen.

### 3.9. Anti-Chelating Agent

Heavy metals are divided into two categories based on their toxicity: essential heavy metals and non-essential heavy metals. Essential heavy metals are harmless or relatively less harmless at low concentrations, such as zinc, copper, iron, and cobalt. However, when the accumulation of the same elements is higher than the threshold, they can cause toxicity, whereas non-essential metals are highly toxic, even at a low concentration, such as cadmium, mercury, arsenic, and chromium [157]. Heavy metal accumulation in the human body severely damages different organs, including the respiratory, nervous, and reproductive systems, as well as the digestive tract [158,159]. Common chelating agents used as chelators are dimercaprol, dimercaptosuccinic acid, 2,3-Dimercaptopropane-1-sulfonic acid, sodium-calcium edetate, deferoxamine, and penicillamine [157]. However, some patients have deteriorating conditions from these chelating agents. Common side effects reported were fever, nausea, headache, vomiting, irregular blood pressure, gastrointestinal distress, sore muscles, pain at the injection site, and burning sensation.

In worst scenarios, it could also cause heart failure, breathing difficulties, respiratory failure, low blood pressure, irreversible kidney damage, convulsions, and low blood calcium [160,161,162]. Hence, researchers are searching for new antidotes sourced from natural sources with higher treatment efficacy with fewer side effects. A recent in vivo study by Daud and others tested an aqueous extract from *C. lentillifera* against lead accumulation in internal tissues of male rats [122]. With *C. lentillifera* being rich in antioxidants, they hypothesised that it might be a good candidate as a chelating agent, as administration of antioxidants has been reported to have protective effects against heavy metal-induced tissue damage. From their experiment, lead intoxicated rats treated with the seaweed extract had significantly higher body weight compared to lead-acetate treated rats, which indicated the capability of the extract to reduce the ill effects. The lead accumulation levels in the blood and internal organs among the intoxicated rats were also reduced.

### 3.10. Immunostimulatory

The innate immune system is one of the physiological defence mechanisms that identifies and eliminates foreign substances while maintaining immune homeostasis via mechanisms that compete with cell proliferation and death [163]. The immune system protects organs from pathogens and antigens, and the development of natural, non-toxic immunomodulators to enhance the immune regulatory system is more effective for long-term health care [125]. Recent studies showed that *C. lentillifera* possesses potential and potent immunomodulatory capabilities [123,124,125,126]. An in vitro experiment conducted by Maeda and colleagues, sulfated polysaccharides from *C. lentillifera* were found to activate and promote the growth of murine macrophages RAW 264.7 cells after 24 h of incubation, with a dosage concentration of 1–5 µg/mL. In addition, the data showed that the secretion of IL-6, TNF-α, and IL-1β was promoted after incubation with *C. lentillifera* polysaccharides, and the functions of CLP were like those of lipopolysaccharides [123]. 

Sun and others assessed the potential as a natural immunomodulator of four novel purified polysaccharides (CLGP1, CLGP2, CLGP3, CLGP4), suggesting a type of xyloglactomannan. All polysaccharides demonstrated immunostimulatory activity at concentrations of 50 to 800 µg/mL, which stimulated the viability of RAW264.7 cells, phagocytic activity, production of nitrite, and acid phosphatase signal enzyme. They concluded that CLGP4 showed the most potent immunostimulatory activities among all polysaccharides [124]. Similar outcomes were observed in another in vitro experiment using RAW 264.7 cells as the study model [125]. In another study, an in vivo experiment was conducted using polysaccharides from *C. lentillifera* that underwent in vitro fermentation using cytoxan (CTX)-induced immunosuppressed BALB/c mice. At concentrations of 25, 50, and 100 mg/kg of *C. lentillifera* polysaccharides, a significant increase in short-chain fatty acids concentrations and regulated the diversity and composition of gut microbiota were observed in immunocompromised BALB/c mice. This results in improved immunostimulatory effects against CTX-induced immunosuppression, including repairing body weight, colon length, and thymus/spleen indexes, and stimulating the production of IL-1, TNF-α, secretory immunoglobulin A, mucin2, and superoxidase dismutase. These findings indicate that *C. lentillifera* can act as microbiota regulators in the gut, potentially improving the immune system in immunocompromised mouse models.

## 4. Methodology

The information was electronically retrieved through various online databases (Scopus, ScienceDirect, Google Scholar, PubMed, etc.) from 2002 to 2022. Using the primary search phrase "*Caulerpa lentillifera*", a total of 138 records were found. Upon screening by applying other relevant keywords such as "nutrient content", “nutritional value”, “antioxidant”, and “health benefits” to obtain relevant journal articles with valuable data inputs, a total of 50 papers were selected. In this review, the data on nutrient composition and reported health benefits were obtained only from journal articles written in English, excluding review articles and conference papers. Data from organizations such as the World Health Organization, the Ministry of Health Malaysia, and the European Food Safety Authority were also adopted in this review paper.

## 5. Conclusions

In this review, the nutrient composition of *C. lentillifera* was compiled. This included carbohydrates and fibre, proteins and amino acids, lipids and fatty acids, minerals, vitamins, pigments, and antioxidant profiles. Health benefits contributed by *C. lentillifera* reported in past studies, such as cardioprotective properties (i. e., anti-hypertensive and hypolipidemic), antibacterial, anticancer, anti-coagulant, anti-hyperglycaemic, anti-diabetic, anti-inflammatory, antioxidative, anti-pyretic, chelating agent, and immunostimulatory, were also described and discussed. Despite the excellent nutrient profile of *C. lentillifera,* it is still underutilised and only wildly cultivated globally. In the future, we hope that more studies on functional food development and cultivation techniques concerning *C. lentillifera* will be conducted, as it could be a solution for food and nutrient security problems around the world. Furthermore, extensive studies on the isolates and extracts from *C. lentillifera* are extremely important. They are needed to understand its bioactivity and mechanisms of action while highlighting its commercialization potential, especially for nutraceutical and pharmaceutical uses.

## Figures and Tables

**Table 1:** Global seaweed production and comparison by region in 2019 [2].

Countries/Region	Total Seaweed Production (Farmed and Wild)		Seaweed Cultivation	
	Tonnes (Wet wt.)	Share of World Production (%)	Tonnes (Wet wt.)	Share in Farmed and Wild Production (%)
**World**	**35,762,504**	**100.00**	**34,679,134**	**96.97**
**Asia**	**34,826,750**	**97.38**	**34,513,223**	**99.10**
1. China	20,296,592	56.75	20,122,142	99.14
2. Indonesia	20,296,592	56.75	20,122,142	99.14
3. Republic of Korea	1,821,475	5.09	1,812,765	99.52
4. Philippines	1,500,326	4.20	1,499,961	99.98
5. DPR of Korea	603,000	1.69	603,000	100.00
7. Japan	412,300	1.15	345,500	83.80
8. Malaysia	188,110	0.53	188,110	100.00
**America**	**487,241**	**1.36**	**22,856**	**4.69**
6. Chile	426,605	1.19	21,679	5.08
**Europe**	**287,033**	**0.80**	**11,125**	**3.88**
9. Norway	163,197	0.46	117	0.07
**Africa**	**144,909**	**0.41**	**117,791**	**81.29**
10. United Republic of Tanzania	106,069	0.30	106,069	100.00
**Oceania**	**16,572**	**0.05**	**14,140**	**85.32**

Data from FAO Fishery and Aquaculture Statistics. Global Aquaculture Production 1950–2019 (FishStatJ).

**Table 2:** Proximate composition and fibre contents of *C. lentillifera* from different countries.

	China	Indonesia	Malaysia	Philippines	Taiwan	Thailand	USA	Vietnam	Reference
Water content, % ^a^	95.09–95.95	77.57–95.01	87.05–92.3	90.1–91.7	94.28	95.4–95.8	94	-	[20,21,22,23,24,25,26,27,28,29,30,31,32,33]
Ash, % dw	25.31–55.20	1.02–3.41	2.1–29.61	4.17–26.57	1.27–22.2	24.21–57.01	46.4	-	[20,21,22,23,33,34,35,36]
Moisture, % ^b^	12.91–13.66	-	-	-	6.42	25.31		16	[33,36,37]
Carbohydrate, % dw	21.32–50.71	0.36–17.08	44.02–72.9	61.82	3.67–69.75	59.27	11.8	44	[20,22,23,24,25,26,27,29,30,31,32,33,34,35,36,37]
Protein, % dw	12.5–14.76	0.43–3.84	13.24–19.38	0.78–5.1	0.53–10.5	4.67–12.49	9.7	4.89–7.0	[20,22,23,24,25,26,27,28,29,30,31,32,33,34,35,36,37]
Lipid, % dw	0.78–2.32	0.32–0.79	0.7–2.87	0.05–0.75	0.09–1.57	0.86–2.0	7.2	1.2–14.0	[20,22,23,24,25,26,27,28,29,30,31,32,33,34,35,36,37]
Fibre, % dw	7.81–12.98	14.38	4.12–19.4	-	0.17–2.97	-	-		[23,24,25,26,27,29,32,33]
Total dietary fibre, g/100 g	33.44–37.16	-	32.99	30.67	-	-		17.5	[25,33,34,36,37]
Insoluble fibre	26.56–28.98	-	15.78	27.17	-	-	-	16.6	[31,32,33,36,37]
Soluble fibre	2.45–8.6	-	17.21	3.5	-	-	-	2.6–4.21	[24,33,34,35,36,37]

^a^ Wet weight; ^b^ Dry weight.

**Table 3:** Amino acid profile of *C. lentillifera*.

Amino Acids	g/100 g Sample	Mean ± SD	Reference
*Essential amino acids*	g/100 g		[31,32,33]
Threonine	0.79–9.3	4.94 ± 3.49	
Valine	0.87–11.16	5.66 ± 4.09	
Lysine	0.68–7.78	4.59 ± 3.16	
Histidine	0.08–2.07	0.98 ± 0.81	
Isoleucine	0.62–6.94	3.71 ± 2.57	
Leucine	0.99–12.86	6.51 ± 4.68	
Methionine	0.18–2.37	1.45 ± 0.93	
Phenylalanine	0.61–6.6	3.57± 2.38	
Total EAA	4.7–57.01	29.86 ± 21.10	
*Non-essential amino acids*			
Aspartic acid	1.43–14.89	8.37 ± 5.74	
Serine	0.76–9.47	5.00 ± 3.60	
Cysteine	0.81–1.2	1.03 ± 0.18	
Glutamic acid	1.77–14.72	9.30 ± 6.15	
Glycine	0.64–19.23	9.17 ± 8.14	
Arginine	0.83–6.21	3.86 ± 2.56	
Alanine	0.85–13.36	6.57 ± 5.07	
Tyrosine	0.48–4.74	2.65 ± 1.78	
Proline	0.57–5.75	3.43 ± 2.34	
Total NEAA	7.67–90.0	49.67 ± 35.45	
Total amino acids	12.37–147.0	63.84 ± 59.40	
EAA/NEAA	0.61–0.63: 1		

**Table 4:** Mineral element composition in *C. lentillifera* in different countries.

Countries	Australia	China	Malaysia	Philippines	Thailand	USA	Vietnam	Reference
Element								
Aluminium, Al	-	8.57	-	-	-	-	744	[32,36]
Antimony, Sb	-	3.25–4.18	-	-	-	-	-	[32]
Arsenic, As	1.06 µg/g	5.14–6.46	-	-	-	-	≤1	[21,32,36]
Barium, Ba	-	0.26–1.71	-	-	-	-	4.75	[32,36]
Beryllium, Be	-	0.38–1.71	-	-	-	-	-	[32]
Boron, B	18.4 µg/g	2.37–2.58	-	-	-	70 µg/g	21.7	[20,21,32,36]
Cadmium, Cd	0.53 µg/g	0.36–0.7	-	-	-	-	1.14	[21,32,36]
Calcium, Ca	16,650 µg/g	0.77–3728.35	32.7–118.66	988.44	780	0.0095	8137	[20,21,22,23,32,33,35,59]
Cerium, Ce	-	0.83–1.04	-	-	-	-	-	[32]
Chromium, Cr	-	0.23–0.34	-	-	-	-	3.3	[32,36]
Cobalt, Co	-	0.03–0.07	-	-	-	-	1.35	[32,36]
Copper, Cu	0.89 µg/g	3.04–20.37	1.18–3.0	-	2200 µg/g	1 µg/g	2.74	[20,21,22,23,32,33,36,59]
Gallium, Ga	-	0.11–0.15	-	-	-	-	-	[32]
Iodine, I	-	0.73–26.3	4.78 µg/g	-	1424 µg/g	-	-	[24,32]
Iron, Fe	-	13.62–1972.97	145.0	430.93	9.3	167 µg/g	595	[20,23,32,33,35,36]
Lithium, Li	-	0.28–2.15	-	-	-	-	-	[32]
Magnesium, Mg	5.875 mg/g	1.93–8126.59	78.33–170.0	-	630	0.0165	10,663	[21,23,32,33,36,59]
Manganese, Mn	-	5.54–1341.07	-	-	7.9	10 µg/g	425	[20,32,33,36]
Molybdenum, Mo	-	0.02–0.05	-	-	-	-	1.32	[32,36]
Nickel, Ni	-	-	-	-	-	-	1.88	[36]
Nitrogen, N	-	0.18–1.10	-	-	-	0.0239	-	[20,32]
Phosphorus, P	-	-	11.22–25.40	-	1030	0.0016	1073	[20,23,33,36,59]
Lead, Pb	0.16 µg/g	-	-	-	-	-	-	[21]
Potassium, K	-	0.91–4967.34	66.16–1413.0	-	970	0.007	1066	[20,23,32,33,36,59]
Rubidium, Rb	-	2.24–2.57	-	-	-	-	-	[32]
Selenium, Se	3.9 µg/g	0.02–0.05	-	-	-	-	≤1	[21,32,39]
Sodium, Na	-	14.90–9432.33	933.83–12,297.0	-	-	-	130,794	[23,32,36,59]
Strontium, Sr	143 µg/g	10.19–11.31	-	-	-	-	104	[21,32,36]
Sulphur, S	-	-	-	-	-	0.0155	6733	[36]
Tin, Sn	-	0.021–0.024	-	-	-	-	-	[32]
Titanium, Ti	-	0.07–0.16	-	-	-	-	-	[32]
Vanadium, V	0.44 µg/g	0.07–0.32	-	-	-	-	2.46	[21,32,36]
Zinc, Zn	27.55 µg/g	1.89–33.90	0.14–6.2	1.09	2.6	17 µg/g	15.2	[20,21,23,32,33,35,36,59]

All values are presented in mg/100 g sample unless stated otherwise.

**Table 5:** Fatty acids composition in *C. lentillifera*.

	Fatty Acids, %	Reference
*Saturated fatty acids*		
C 3:0	15.92	[35]
C 4:0	2.3	[26]
C 6:0	0.002–0.3	[26,30,32]
C 8:0	0.0004–1.1	
C 10:0	0.0001–6.4	[24,26,30,32]
C 11:0	0.85–1.1	[24,26,32]
C 12:0	0.006–0.69	[24,26,30,32]
C 13:0	0.001–1.54	[24,26,30,32]
C 14:0	0.019–2.92	[24,26,30,32]
C 15:0	0.001–2.1	[24,26,30,32]
C 16:0	0.22–49.46	[24,26,30,33]
C 17:0	0.0001–3.36	[24,26,30,32]
C 18:0	0.012–7.83	[24,26,30,33]
C 20:0	0.001–1.98	[24,26,30,33]
C 21:0	0.001–1.62	[24,26,30,32]
C 22:0	0.005–1.15	[24,26,30,33]
C 23:0	0.01–2.05	[24,26,30,32]
C 24:0	0.041–8.85	[24,26,30,32]
*Monounsaturated fatty acids*		
C 14:1	0.001–1.5	[24,26,30,32]
C 14:1 ω-9	0.59	[31]
C 15:1	0.83–2.54	[26,31,32]
C 16:1 ω-9	0.029–8.24	[33]
C 17:1	0.0003–2.67	[24,26,32]
C 18:1 ω-9c	0.03–32.49	[24,26,30,32]
C 18:1 ω-9t	0.22–0.93	[24,26,30,32]
C 20:1	0.18–1.69	[26,33]
C 20:1 ω-9	0.009–0.17	[24]
C 22:1 ω-9	0.0001–2.8	[24,26,33]
C 24:1 ω-7	0.1–2.79	[26,32]
C 24:1 ω-9	0.66–0.93	[24,30]
*Polyunsaturated fatty acids*		
**n-6 PUFA**		
C 18:2 (ω6c)	0.48–13.14	[30,32]
C 18:2 (ω6t)	0.09–4.13	[33]
C 18:3 (ω6)	0.002–13.89	[24,33]
C 20:2 (ω6)	0.002–4.27	[24,30]
C 20:3 (ω6)	0.001–3.3	[32]
C 20:4 (ω6)	0.003–6.7	[33]
C 22:2 (ω6)	0.95–1.56	[30]
C 22:6 (ω6)	0.11–0.83	[32,33]
**n-3 PUFA**		
C 18:3 (ω3)	0.035–13.30	[24,32]
C 20:3 (ω3)	0.001–2.72	[24,32]
C 20:5 (ω3)	0.003–1.91	[24,33]
C 22:6 (ω3)	0.003–3.64	[24,30,33]

**Table 6:** Vitamin content in *C. lentillifera*, the daily recommended nutrient intake (RNI), and the tolerable upper intake level (U.L.) per day.

Vitamins	Present in *C. lentillifera*	RNI/Day ^1^	UL/day ^2^	Reference
Thiamine (Vitamin B1), mg/100 g	0.021–8.8	1.1–1.2 mg	ND	[22,23]
Riboflavin (Vitamin B2), mg/100 g	0.02–2.5	1.1–1.3 mg	ND	
Vitamin B3 (as niacin), mg/100 g	1.09–200	14–16 mg NE	35 mg NE	
Vitamin C, mg/100 g	0.028–274	70 mg	2000 mg	[8,22,24,32,33]
Vitamin E, mg α-tocopherol/g	0.02–0.46	7.5–10 mg	1000 mg	[24,32,33]
Vitamin A (as 𝛽-carotene), µg RE/g	0.1–1530	600 µg RE	3000 µg RE	[8,20,21,22,23,24]

^1^ The values of RNI per day are for adults aged 19–65. ^2^ RNI and tolerable upper intake levels are obtained from Recommended Nutrient Intakes for Malaysia (Ministry of Health, 2017).

**Table 7:** The concentration of pigments found in *Caulerpa lentillifera*.

Pigments	Concentration (mg/100 g)	Reference
Chlorophylls	0.729–82.32	[22,37,80]
Chlorophyll a	0.332–53.0	
Chlorophyll b	0.397–118.0	
Carotenoids	2.578–22.0	[22,80]
Astaxanthin	3.0	[77]
𝛽-Carotene/Lycopene	0.1–1530.0 µg RE/g	[20,21,24,77,80]
Caulerpin	25.79–33.59 μg/g	[37]
𝛽-Cryptoxanthin	1.3	[77]
Canthaxanthin	14.6	
Fucoxanthin	<0.001	
Lutein	<0.02–2.113	[77,80]
Violaxanthin	0.893	[80]
Zeaxanthin	0.213–3.6	[77,80]

**Table 9:** Antioxidant activities in *Caulerpa lentillifera*.

Solvent	*ETN	AC	RA	TPC, mg GAE/g	TFC, mg QE/g	DPPH, %	FRAP, mg TE/g	TEAC, %	H_2_O_2_ activity, %	ORAC, μmol T.E./100 g	Reference
		mg AAE/g	mg FESO_4_/g								
Methanol	0.762			42.85			362.11	2.16 ^b^			[150]
				51.87							[24]
				52.85							[25]
				4.52	4.93	9.74					[111]
				16.8–28.56							[29]
				0.58	15.41	2.87	0.27				[59]
Ethanol	0.654			1.3–2.04		1.21–31.68		0.08–46.46	88.78–94.81		[8]
		54.23–79.09 ^c^									[28]
						5.74				68,372	[77]
		21.19–26.37	17.92–21.34	6.23–7.28 ^a^		63.19–73.2					[37]
											[151]
Water	1			2.04	1.17	81.55					[108]
						3.04		18.26–29.3			[151]
Acetone: Hexane	NA					47					[80]
Butanol	0.586					4.5–11.0		21.99–22.17			[151]
Chloroform	0.259			5.47	0.28	2.2					[111]
Ethyl acetate	0.228					25.64–91.25		28.75–84.37			[151]
Hexane	0.009					13.97–38.27		9.7–40.41			[151]

*ETN: solvent polarity index ^a^ Expressed in mg phloroglucinol (PGE) per gram dry extract; ^b^ Expressed in μM/mg dw; c Expressed in %.

## Data Availability

Not applicable.
